# Evaluation of the image quality and validity of handheld echocardiography for stroke volume and left ventricular ejection fraction quantification: a method comparison study

**DOI:** 10.1007/s10554-023-02942-7

**Published:** 2023-10-10

**Authors:** Frederique M. de Raat, Joris van Houte, Leon. J. Montenij, Sjoerd Bouwmeester, Suzanne E. A. Felix, Peter Bingley, Esmée C. de Boer, Patrick Houthuizen, Arthur R. Bouwman

**Affiliations:** 1 Department of Anesthesiology, Catharina Hospital, Eindhoven, The Netherlands; 2 Department of Cardiology, Catharina Hospital, Eindhoven, The Netherlands; 3 Department of Electrical Engineering, Technical University of Eindhoven, De Zaale, Eindhoven, The Netherlands; 4 Department of Patient Care & Measurements, Philips Research, Eindhoven, The Netherlands

**Keywords:** Handheld echocardiography, Hemodynamic monitoring, Left ventricle ejection fraction, Point of care ultrasound, Stroke volume

## Abstract

Bedside quantification of stroke volume (SV) and left ventricular ejection fraction (LVEF) is valuable in hemodynamically compromised patients. Miniaturized handheld ultrasound (HAND) devices are now available for clinical use. However, the performance level of HAND devices for quantified cardiac assessment is yet unknown. The aim of this study was to compare the validity of HAND measurements with standard echocardiography (SE) and three-dimensional echocardiography (3DE). Thirty-six patients were scanned with HAND, SE and 3DE. LVEF and SV quantification was done with automated software for the HAND, SE and 3DE dataset. The image quality of HAND and SE was evaluated by scoring segmental endocardial border delineation (2 = good, 1 = poor, 0 = invisible). LVEF and SV of HAND was evaluated against SE and 3DE using correlation and Bland–Altman analysis. The correlation, bias, and limits of agreement (LOA) between HAND and SE were 0.68 [0.46:0.83], 1.60% [− 2.18:5.38], and 8.84% [− 9.79:12.99] for LVEF, and 0.91 [0.84:0.96], 1.32 ml [− 0.36:4.01], 15.54 ml [− 18.70:21.35] for SV, respectively. Correlation, bias, and LOA between HAND and 3DE were 0.55 [0.6:0.74], − 0.56% [− 2.27:1.1], and 9.88% [− 13.29:12.17] for LVEF, and 0.79 [0.62:0.89], 6.78 ml [2.34:11.21], 12.14 ml [− 26.32:39.87] for SV, respectively. The image quality scores were 9.42 ± 2.0 for the apical four chamber views of the HAND dataset and 10.49 ± 1.7 for the SE dataset and (*P* < 0.001). Clinically acceptable accuracy, precision, and image quality was demonstrated for HAND measurements compared to SE. In comparison to 3DE, HAND showed a clinically acceptable accuracy and precision for LVEF quantification.

## Background

In critically ill patients, the assessment of cardiac function is important in the early diagnosis and treatment of hemodynamic and respiratory instability. Evaluation of cardiac function supports the clinician in appropriate decision making in terms of fluid therapy, and vasoactive or inotropic support [1, 2]. To this end, bedside cardiac ultrasound is increasingly used in the emergency department, intensive care unit and perioperative setting for non-invasive quantification of left ventricular ejection fraction (LVEF) and stroke volume (SV) [3].

Two-dimensional (2D) standard echocardiography (SE) using a high-end ultrasound system is still considered the preferred clinical method. Three-dimensional echocardiography (3DE) has additional benefits such as a reduction of geometric assumptions and therefore became an integral part of the echocardiography landscape [4]. However, the limited availability, high costs, poor transportability and flexibility of echo machines capable of 2- and 3-dimensional imaging often hinder a high level of accessibility at the bedside.

Over the past years, technological advancements have resulted in the emergence of miniaturized handheld ultrasound (HAND) devices that are compact and battery operated. Their simplicity of use, pocket size shape and therefore high portability, and easy connectivity to a mobile device serves both comfort and convenience for the treating physician. Caregivers may therefore feel encouraged to use such devices for point of care medical decision making, and it was shown earlier that the use of HAND is rapidly increasing among intensivists, emergency care physicians, and anesthesiologists [5].

Several studies have investigated the diagnostic accuracy of HAND for the evaluation of volume status, pericardial effusions or valve abnormalities [6–8]. However, literature lacks a comprehensive analysis of the validity of HAND for automatic objective LVEF and SV quantification. Furthermore, the quantification of the image quality of HAND to assess cardiac function has not been addressed before. In this cross-sectional observational study, we aimed to evaluate the validity and image quality of a commercially available HAND device to quantify LVEF, SV with an automatic tool in non-hospitalized cardiac patients. It was hypothesized that SV and LVEF measurements from HAND are interchangeable with SE but not with 3DE, as 3DE measurements of cardiac chamber volumes do not rely on geometric assumptions about their shape, and are closer to values provided by cardiac magnetic resonance than 2D volumes [9]. With this study we intended to provide more clinical insights in the baseline performance characteristics of HAND for future point of care applicability.

## Methods

This prospective cross-sectional observational study was conducted from January 2021 till May 2022 at the Cardiology department of the Catharina hospital, Eindhoven, the Netherlands. Patients who needed standard of care transthoracic echocardiography were sent to the echo lab of the Catharina Hospital by their referring cardiologist. Exclusion criteria were age below 18 years, poor delineation of the endocardial border on SE images, (supra)ventricular arrhythmias, moderate to severe valvular disease, and moderate to severe pulmonary hypertension. Written informed consent was obtained from all patients. This study (W21.051) was approved by the institutional review board of the Medical Ethical Centre of Utrecht the Netherlands and carried out in accordance with the declaration of Helsinki.

### Data acquisition

A dedicated expert cardiologist blinded to the post-processing results conducted all acquisitions. Patients were breathing spontaneously and were placed in left lateral position at the time of examination. During image acquisition patients were asked to perform an expiratory hold maneuverer. Gain, focus, and depth settings were adjusted to maximize endocardial visualization. Three different echo modalities (SE, 3DE, and HAND) were used for image acquisition in each patient:

SE and 3DE single beat images were acquired with an EPIQ ultrasound system equipped with a X5-1 matrix array transducer (1–5 MHz, Philips Ultrasound, Inc., Bothell, WA). The harmonic function was used to optimize image resolution. Storage and looping of cardiac cycles were ECG triggered. For the SE data set a two-dimensional view of the apical four chamber (A4CH), and apical two chamber (A2CH) view were obtained. For the 3DE dataset a single beat, wide-angled ‘full volume’ 3DE image was acquired from the A4CH view position.

HAND measurements were obtained with the Lumify S4-1 phased array transducer (1–4 MHz, Philips Ultrasound, Inc., Bothell, WA). This device does not support ECG triggered storage and looping of a single cardiac cycle, as it does not have ECG input capability. Instead, 8 s recordings of both the A2CH and A4CH views were acquired. From this dataset, only the second heartbeat was used for analysis.

Data acquisition was done sequentially in the following order: SE, 3DE and HAND. All measurements were performed in triplicate without changing probe position. Total acquisition time was approximately 5–10 min. Images were saved as DICOM files and exported to the hospital server for offline post-processing. Offline post-processing was done by an independent blinded analyst trained in performing echocardiographic measurements.

### Data quantification

For quantification of the HAND and SE dataset the Auto Strain (AS) tool (*TOMTEC—ARENA Lot 50, TOMTEC Imaging Systems GmbH, Germany*) was used. This tool automatically identifies the end-diastolic and end-systolic frames using the ECG signal.

**Fig. 1:**
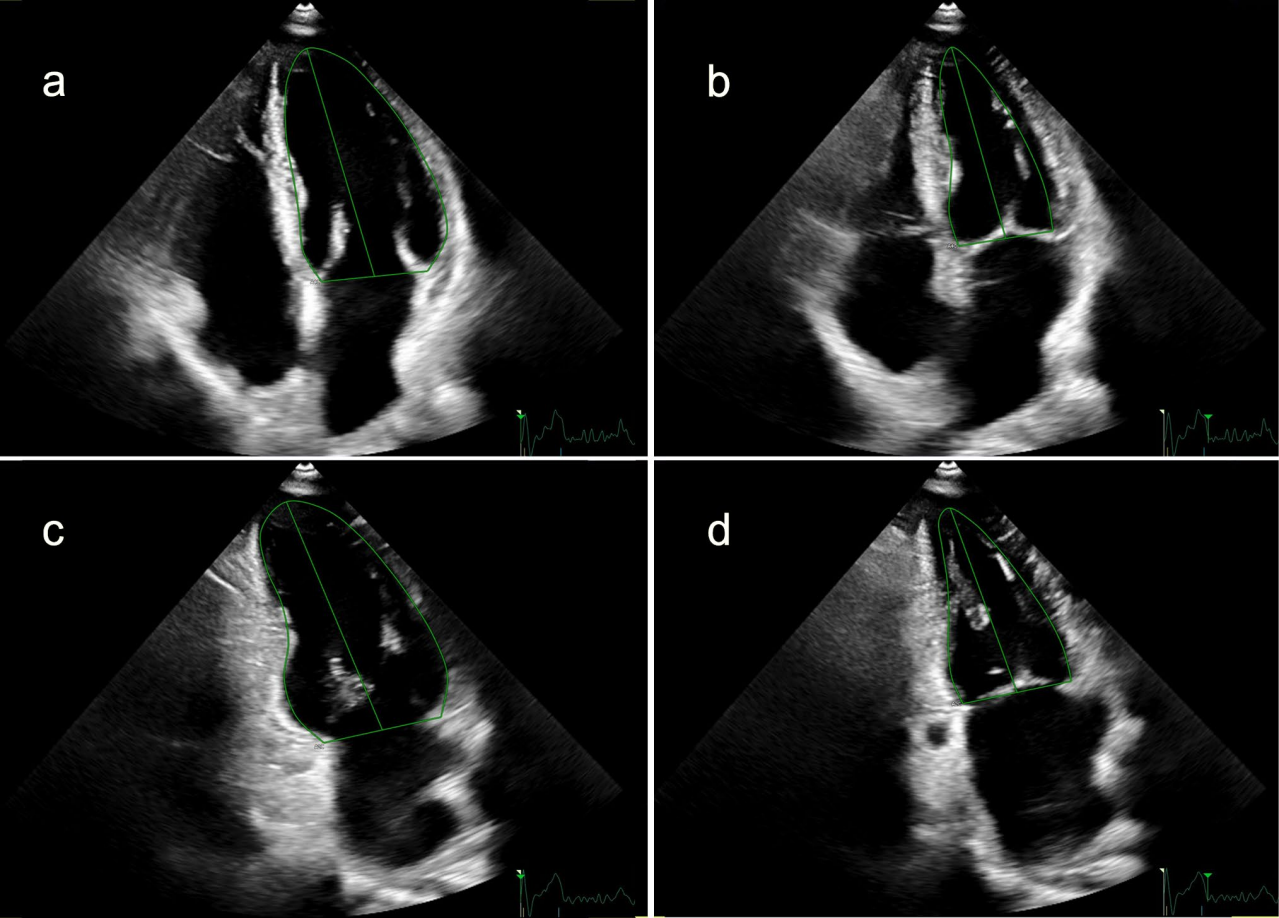
This figure shows the delineation of the left ventricle (green line) throughout the heart cycle in images from the SE dataset. **a** A4CH view in diastole; **b** A4CH view in systole, **c** A2CH in diastole, **d** A2CH in systole

**Fig. 2:**
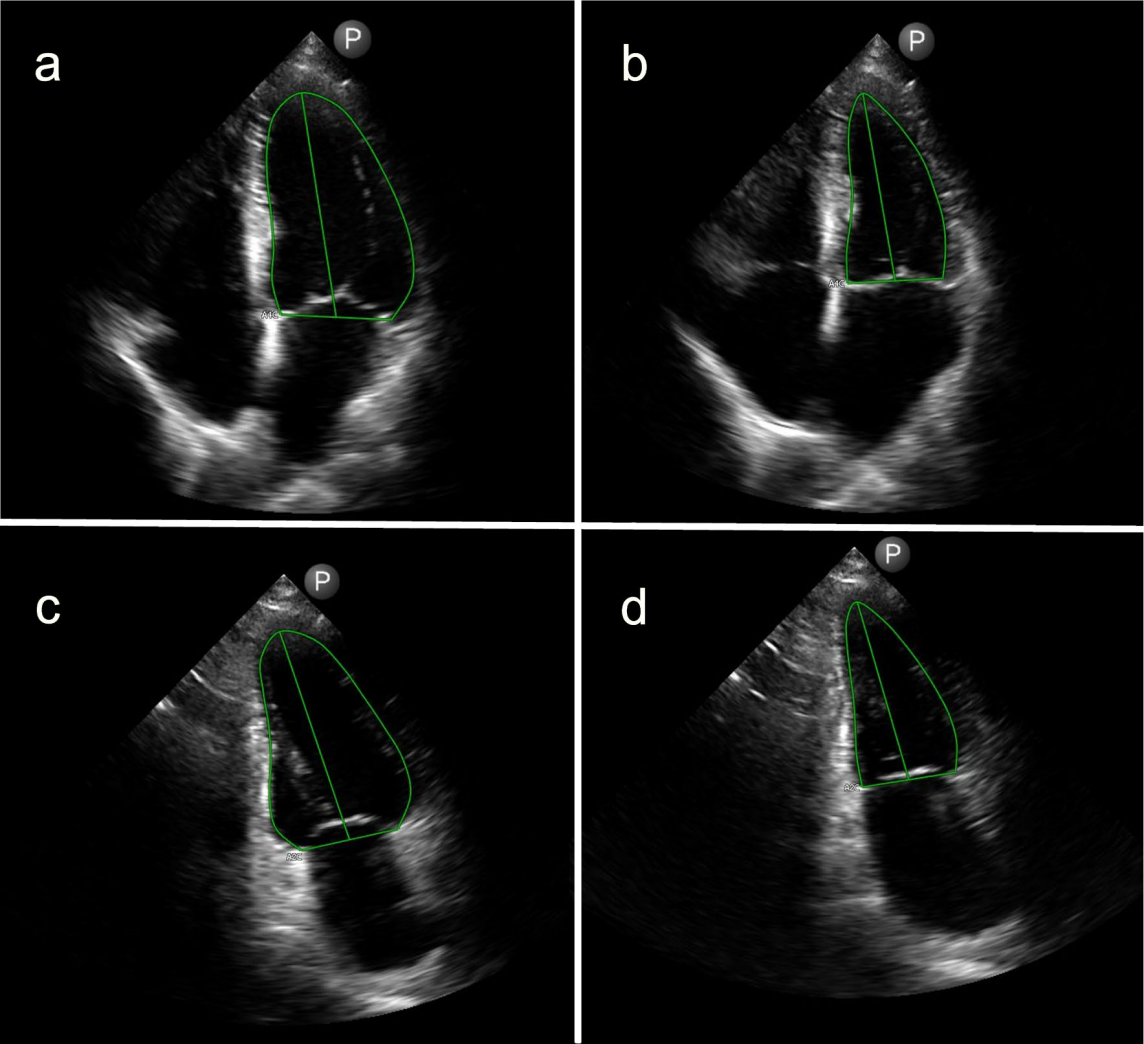
This figure shows the delineation of the left ventricle (green line) throughout the heart cycle in images from the HAND dataset. **a** A4CH view in diastole, **b** A4CH view in systole; **c** A2CH in diastole, **d** A2CH in systole

However, as the HAND dataset did not contain an ECG signal, the end-diastolic and end-systolic frames had to be selected manually using M-mode tracing through the mitral valve annulus. Next, based on the automatically traced LV blood-tissue boundaries (Figs. 1 and 2) and the Simpson’s biplane method, SV and LVEF were calculated.

For 3DE quantification the Dynamic HeartModel^A.I.^ (*DHM, Philips Ultrasound, Inc., Bothell, WA*) was used. This tool automatically identifies the end-diastolic and end-systolic frames from the cardiac cycle using ECG and creates end-diastolic and end-systolic 3D projections of the LV cavity. From these 3D projections LV parameters were derived directly. Manual adjustments to the endocardial border tracings were not supported by DHM. Hence, when the operator judged the automatically detected endocardial borders to be incorrect, those images were excluded from analysis.

### Statistical analysis

A sample size calculation was performed to limit the width of the 95% confidence interval (CI) around the standard deviation (SD) of the bias to 10%. Based on a mean SV of 60 ml and a mean error of 30%, a sample size of 32 patients was calculated to be sufficient [10, 11]. We included 43 patients in order to account for a potential drop-out rate of approximately 25% due to insufficient image quality.

Statistical analysis and data visualization were performed using IBM SPSS statistics (*version 22, IBM Corp, USA*) and MATLAB (*MATLAB 2020a, MathWorks, Inc. USA).* Data are shown as mean ± SD or median [IQR] as appropriate. The assumption of normality was tested using the Shapiro–Wilk normality test.

In this article validity is based on assessment of correlation, accuracy and precision. Correlation calculations were performed using linear regression with Pearson’s correlation coefficient or Spearman’s correlation as appropriate. Correlation coefficients were considered poor (< 0.4), moderate (0.4–0.7), strong (0.7–0.9), or very strong (> 0.9) [12]. To determine the reliability for the quantification of LVEF and SV the intraclass correlation coefficient of agreement (ICC-agreement) was calculated. Additionally, the interclass correlation of agreement between techniques (ICC-techniques) was calculated. ICC-agreement and ICC-techniques values were considered moderate (< 0.75), good (0.76–0.9), or excellent (> 0.9) [13].

**Fig. 3:**
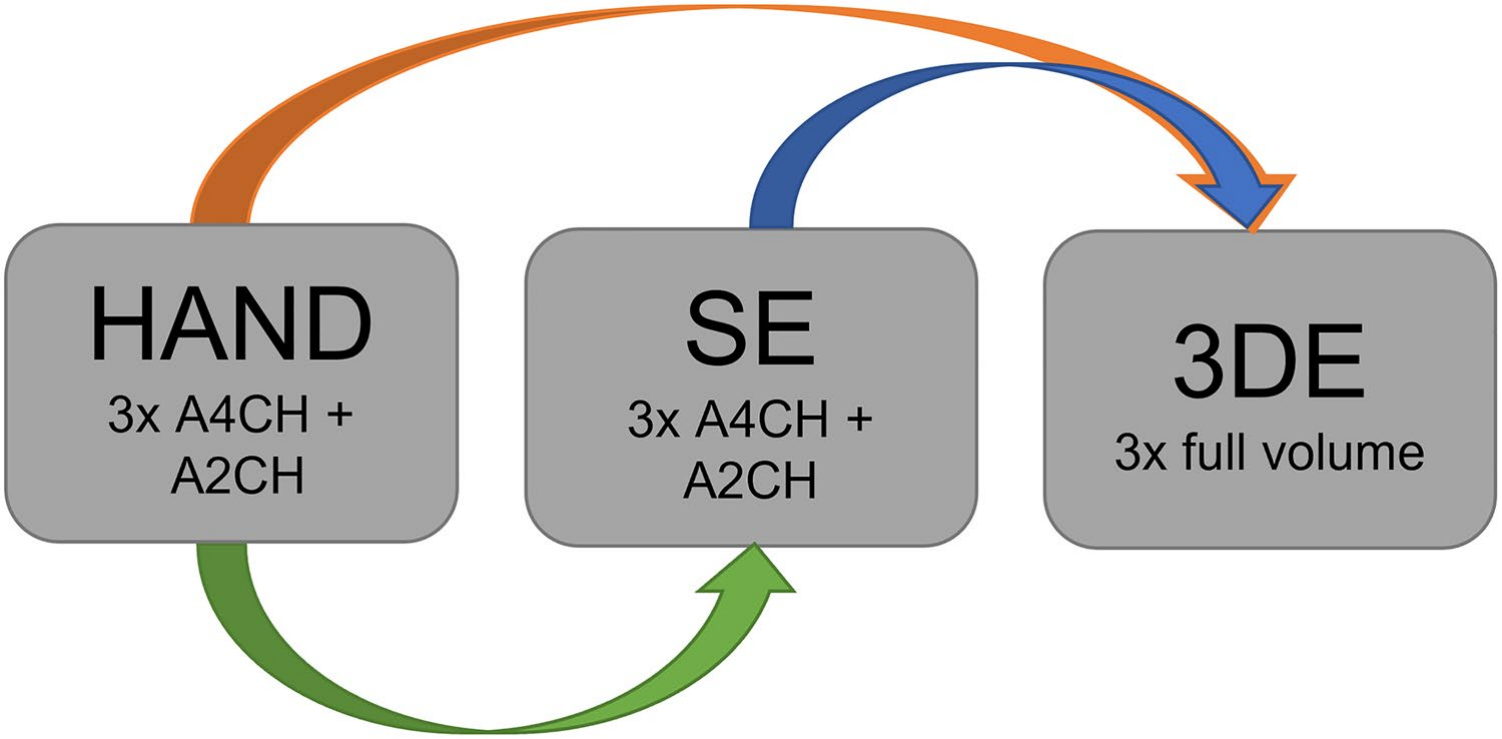
Shows a diagram of the different inter-technique comparisons; 1) HAND vs SE (green arrow); HAND vs 3DE (orange arrow); SE vs 3DE (blue arrow) (Graphics program used: PowerPoint)

Bland–Altman analysis was used to evaluate the agreement between HAND vs SE, HAND vs 3DE and SE vs 3DE (Fig. 3). With the Bland–Altman method the bias is determined as a measure of accuracy, and the 95% limits of agreement (LOA) as a measure of precision. The presence of proportional bias in the Bland–Altman plot was checked with regression analysis. Based on the Bland–Altman and correlation analysis, only conclusions about interchangeability between the experimental technique (HAND) and the reference technique (SE or 3DE) can be drawn. To compare the means of the HAND, SE and 3DE dataset, a two-sided paired samples t-test was performed or a Mann–Whitney U test, depending on normality. P values < 0.001 were considered significant according to the Bonferroni correction. For SV, a bias up to 10% with respect to the mean of the reference method and a mean error up to 30% with respect to the mean of the reference method were considered clinically acceptable [11, 14]. For LVEF, a bias below 10% and a mean error below 15% was considered clinically acceptable [11].

The LOA and mean error are influenced by the precision of the used reference technique, as explained by:

1 $$Meanerror \, (\% ) = \sqrt {experimental \, precision^2 + \, reference \, precision^2 }$$

This emphasizes the need for the evaluation of reference precision in addition to experimental precision. Therefore, the repeatability coefficient (RC) was calculated for HAND, SE and 3DE.

2 $$Repeatability \, coefficient (RC) = 2 \times \sqrt {1,96} \times SD _{within \, subject}$$

To calculate the within-subject SD per tool one-way ANOVA was performed with LVEF and SV values as dependent factors and the subject as independent factor.

Moreover, the coefficient of variation (CV) was calculated for LVEF (CV_LVEF_), and SV (CV_sv_) for each dataset. It is calculated as the ratio between the within-subject SD and the mean. According to literature, a CV below 10% is considered clinically acceptable [15].

Image quality of HAND and SE recordings was evaluated using the seventeen-segment-model from the American Heart Association [16]. As in this study no apical three-chamber views were obtained, only fourteen segments of the model could be assessed (seven in the A4CH and seven segments in the A2CH view). Segmental endocardial border delineation was scored (2 = good, 1 = limited visibility, 0 = invisible) for each segment to quantify the HAND and SE image quality, except for the apical segment which was scored with 0 points or 1 point. The maximal score for the A4CH or A2CH view was 13.

## Results

Fourty-three patients participated in this study, of whom 7 were excluded because of poor endocardial delineation of SE images, and 36 patients were included for analysis. The HAND, SE and 3DE measurements were performed in triplicate in thirty-six, twenty-two and thirty-two patients respectively. For the remaining patients, measurements were performed in duplicate. The mean value of the triple c.q. double measurements per patient are used in the analysis. The baseline characteristics of the patients are presented in Table 1. Endocardial borders were traced correctly in 100% (AS) and 96% (DHM) of the images. Image quality of SE and HAND was significantly different for A4CH images (10.49 ± 1.72 and 9,42 ± 1,96 respectively, p < 0.001) and for A2CH images (9.82 ± 1,99, and 8,49 ± 2,07 respectively, P < 0.001).

**Table 1:** Baseline demographic characteristics

Total number of participants (N)	36
Male (%)	*44*
Age (years)	*55* ± *14*
Body length (cm)	*172.5* ± *8.7*
Body weight (kg)	*76.8* ± *13.5*
BMI (kg/ m^2)^	*25.7* ± *3.8*
Body surface area (m^2^)	*1.9* ± *0.3*
Creatinine (µmol/L)	*82.3* ± *13.8*
Diabetes (%)	*8*
Hypertension (%)	*33*
Myocardial infarction (%)	*8*
Revascularization (%)	*8*
Valvular disease (%)	*0*
Peripheral disease (%)	*11*
COPD (%)	*0*

Values are presented as mean ± SD

*BMI* body mass index, *COPD *chronic obstructive pulmonary disease

**Fig. 4:**
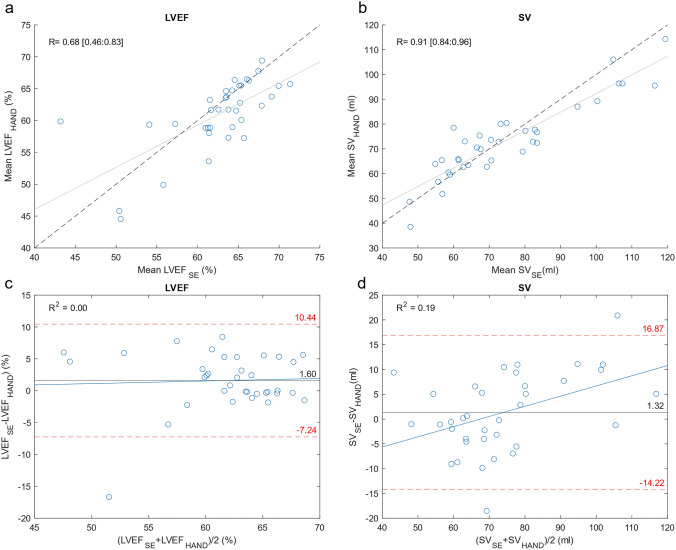
*Upper panel:* Correlation analysis of the HAND and SE data for **a** LVEF, and **b** SV. *Lower panel:* Bland–Altman plots of HAND versus SE for **c** LVEF, and **d** SV. *LVEF* left ventricle ejection fraction, *SV* stroke volume, *R* correlation coefficient, *R*^*2*^ regression coefficient

The correlation between HAND and SE was strong for SV and moderate for LVEF (Table 2, Fig. 4). Accuracy and precision were clinically acceptable for all parameters (Table 2 and Fig. 4).

**Table 2:** Inter-technique comparison—HAND Versus SE

	N	Averaged SE	Averaged HAND	Corr	95% CI of corr	Bias	95% CI of Bias	LOA	95% CI of LOA
LVEF, %									
	*36*	*62.56* ± *5.77*	*60.96* ± *5.57*	0.68*	*[0.46: 0.83]*	*1.60*^*♦*^	*[0.07:3.13]*^*♦*^	*8.84*^*♦*^	*[*− *9.79: 12.99]*^*♦*^
SV, ml									
	*36*	*74.53* ± *5.77*	*73.21* ± *15.61*	0.91*	*[0.83: 0.96]*	*1.32*^*♦*^	*[*−*1.36:4.01]*^*♦*^	*15.54*^*♦*^	*[*− *18.70: 21.35]*^*♦*^

Values are presented as mean ± SD. *p < 0.001

^♦^95% CI within the clinical acceptable LOA or bias range

*Corr* correlation coefficient, *CI *confidence interval, *HAND *handheld ultrasound device, *LVEF* left ventricle ejection fraction, *LOA *limits of agreement, *SE *standard echocardiography, *SV* stroke volume

**Fig. 5:**
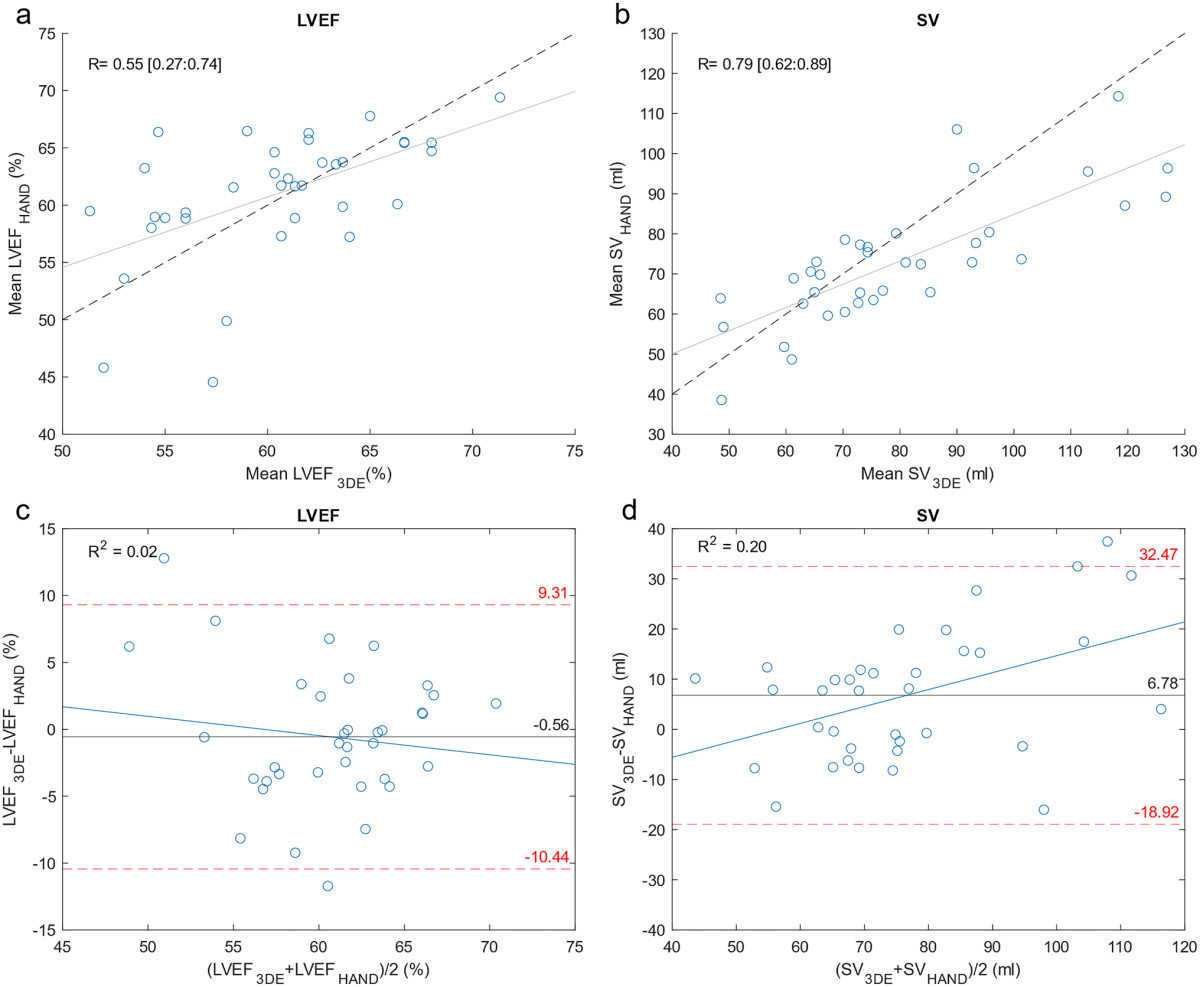
*Upper panel:* Correlation analysis of 3DE and HAND data for **a** LVEF, and **b** SV. *Lower panel*: Bland–Altman plots of 3DE versus HAND for** c** LVEF, and **d** SV. *LVEF* left ventricle ejection fraction, *SV *stroke volume; *R* correlation coefficient, *3DE* three-dimensional echocardiography

The correlation between the HAND and 3DE was strong for SV and moderate for LVEF (Table 3, Fig. 5). Accuracy and precision were clinically acceptable for LVEF. For SV measurements, only accuracy was clinically acceptable (Table 3, Fig. 5).

**Table 3:** Inter-technique quantification method comparison of HAND and SE versus 3DE

	N	Averaged	Averaged 3DE	Corr	95% CI of Corr	Bias	95% CI of Bias	LOA	95% CI of LOA
HAND									
LVEF, %									
	*36*	*60.96* ± *5.57*	*60.39* ± *4.98*	0.55*	*[0.61:0.74]*	− *0.56*	*[*− *2.27:1.14]*^*♦*^	*9.88*	*[*− *13.29: 12.17]*^*♦*^
SV, ml									
	*36*	*73.21* ± *15.61*	*79.98* ± *21.17*	0.79*	*[0.62:0.89]*	*6.78*	*[2.34:11.21]*^*♦*^	*12.14*	*[*− *26.32: 39.87]*
SE									
LVEF, %									
	*36*	*62.56* ± *5.77*	*60.39* ± *4.98*	*0.43*	*[0.12:0.67]*	*− 2.16*	*[*− *4.12:− 0.21]*^*♦*^	*11.31*	*[*− *16.73: 12.40]*^*♦*^
SV, ml									
	*36*	*74.53* ± *5.77*	*79.98* ± *21.17*	0.83*	*[0.69:0.91]*	*5.45*	*[1.46:9.45]*^*♦*^	*23.14*	*[*− *24.38: 35.29]*

Values are mean ± SD. *p < 0.001

^♦^95% CI within the clinical acceptable LOA or bias range

*AS* auto strain, *Corr* correlation coefficient, *CI *confidence interval, *DHM *dynamic heart model, *HAND *handheld ultrasound device, *LVEF *left ventricle ejection fraction, *LOA *limits of agreement, *SE *standard echocardiography, *SV *stroke volume, *3DE* three-dimensional echocardiography

The correlation between the SE and 3DE was strong for SV and moderate for LVEF (Table 3). Accuracy and precision were clinically acceptable for LVEF (Table 3). For SV measurements, only accuracy was clinically acceptable (Table 3). Results of ICC-agreement, RC and CV calculations are presented in Table 4. ICC-agreement, RC and CV were calculated for twenty-two patients in the SE dataset and for thirty-two patients in the 3DE dataset. The results show a comparable intra-reliability and intra-observer repeatability of HAND compared to SE and 3DE according to the ICC-agreement, RC and CV of the datasets. The ICC-techniques shows an excellent reliability of HAND vs SE for SV quantification (Table 5).

**Table 4:** Repeatability coefficient (RC), intraclass correlation coefficient of agreement (ICC-agreement), coefficient of variation (CV)

	N	RC		ICC-agreement		CV	
		SV (ml)	LVEF (%)	SV (ml)	LVEF (%)	SV (ml)	LVEF (%)
HAND	36	*14.76*	*6.75*	*0.950*	*0.908*	*7.24* ± *3.26*	*4.02* ± *2.61*
SE	22	*15.35*	*6.04*	*0.963*	*0.794*	*7.42* ± *4.32*	*3.98* ± *1.78*
3DE	32	*18.62*	*6.49*	*0.962*	*0.921*	*8.08* ± *5.63*	*3.97* ± *2.77*

*CV* coefficient of variation, *ICC-agreement *intraclass correlation coefficient of agreement, *ICC-techniques *interclass correlation coefficient of agreement between techniques, *RC *repeatability coefficient

**Table 5:** Interclass correlation coefficient of agreement between techniques (ICC-techniques)

SV (ml)	HAND	SE	3DE	LVEF (%)	HAND	SE	3DE
HAND	1	*0.945*	*0.830*	HAND	*1*	*0.797*	*0.709*
SE	0.945	*1*	*0.890*	SE	*0.797*	*1*	*0.573*
3DE	0.830	*0.890*	*1*	3DE	*0.709*	*0.573*	*1*

*CV* coefficient of variation, *ICC-agreement *intraclass correlation coefficient of agreement, *ICC-techniques *interclass correlation coefficient of agreement between techniques, *RC *repeatability coefficient

## Discussion

This study investigated the validity and image quality of a handheld echocardiography device versus high-end 2- and 3-dimensional ultrasound reference modalities. The comparison between HAND and SE shows comparable image quality and a clinically acceptable accuracy and precision for SV and LVEF quantification. The correlation was strong for SV quantification and moderate for LVEF. The comparison between HAND and 3DE shows a clinically acceptable accuracy and precision for LVEF but not for SV.

SE is routinely used to evaluate left ventricular function and volume status non-invasively. However, clinical practice could benefit from a technique that is prompt available at the bedside of the patients and can quantify cardiac function objectively. Therefore, this study provides a comprehensive analysis of the validity of HAND for LVEF and SV quantification with an automatic tool. This study demonstrates that LVEF and SV as derived by HAND and SE are interchangeable, which is in line with a previous study that showed strong correlation and agreement between HAND and SE for LVEF quantification [17]. Our results show only a moderate correlation between HAND and SE for LVEF. This is likely due to the fact that the LVEF values encompass a smaller range than the SV values [18]. In addition, the ICC-techniques between HAND and SE show an excellent reliability of HAND compared to SE. In the study of Dustin and colleagues the comparison of HAND with SE showed a wider bias and LOA compared to our study [19]. This can be the result of technological advancements of handheld ultrasound in the past couple of years.

Regarding the comparison of HAND with 3DE, the results showed acceptable agreement for LVEF quantification, while there was no agreement for SV. SV as determined by HAND or SE was structurally underestimated compared with 3DE. The lack of interchangeability between HAND and 3DE for SV quantification could not be explained by the impreciseness of 3DE as reference technique following from formula 1 and the RC of 3DE.

The underestimation of SV with HAND and SE is in concordance with a previously published meta-analysis, in which it was found that 2D ultrasound techniques underestimate LV volumes compared to 3D ultrasound [20]. Therefore, the lack of interchangeability of HAND with 3DE can be explained by the inherent difference between 2 and 3D ultrasound [9].

For clinicians to be able to assess cardiac function through eyeballing, or even better, through an operator independent tool to quantify LVEF or SV, the visibility of the endocardial border is the principal component. Therefore, in this study the main focus of image quality assessment was endocardial border delineation of the LV. The width of the HAND transducer was 3 mm larger compared to the transducer used with SE, which could hinder appropriate acquisition of transthoracic views. On average 2 of 7 endocardial border segments were not visible in the HAND images against 1 of 7 using SE. Since most automatic quantification tools can cope with poor visibility of 2 to 3 endocardial border segments, image quality of HAND recordings should not limit the performance of artificial intelligence driven quantification tools such as the one used in this study.

For quick and operator independent cardiac function assessment the automated quantification tool (AS) in combination with HAND demonstrated to be the preferred method for point of care echocardiography. However, lacking of an imbedded ECG-signal, as in the HAND device in this study, may compromise repeatability and accuracy of automated quantification, as end-diastolic and end-systolic frames still need to be selected manually [21]. This emphasizes the clinical need of an automated tablet-based quantification tool.

This study provides a starting point for future research evaluating the clinical applicability of HAND devices for point of care assessment in a critical care setting. However, the change in SV over time can be more interesting than its absolute value, as dynamic parameters in response to hemodynamic changes have shown to be more valuable in fluid management and hemodynamic support in critically ill patients and during (cardiac) surgery [22, 23]. Therefore, future research should focus on the evaluation of trending ability of hemodynamic parameters derived from handheld devices. Furthermore, this study intended to provide knowledge as a starting point for future point of care application of HAND. This study evaluated the validity of HAND in relatively healthy, and slim patients, who were ideally positioned and breathing comfortably, which allowed for obtaining optimal acoustic windows and therefore adequate visibility of the endocardial border. It would be interesting to evaluate the performance of HAND in a study population of critically ill patients or in a peri-operative setting, in which several conditions may be suboptimal. In addition to this, expert sonographers conducted all examinations, giving rise to a high reproducibility rate and high image quality. Reproducibility and image quality may decrease with less experienced sonographers. Finally, usage of handheld devices should be investigated in patients who are mechanically ventilated to evaluate how this affects performance and image quality [24].

Our study has several limitations. First, the predefined acceptable range for bias (10%) and mean error (15% or 30%), while generally clinically accepted, are a matter of discussion. In this study they are defined as acceptable based on the clear advantages of HAND in comparison to the reference techniques (SE, 3DE) and literature [14]. Depending on the clinical situation, such threshold limits could be adjusted [25, 26]. Second, automated quantification of HAND derived images is M-mode dependent, which is yet a significant limitation for point of care assessment. Third, the results obtained in this study cannot be transferred to patients with low LVEF since they were not included in this study. Finally, cardiac magnetic resonance is currently considered as the gold standard in evaluating cardiac chamber volumes and is therefore an interesting reference for future studies. However, by using 3D transthoracic echocardiography as the reference modality, we were able to compare different echo modalities at the same time frame and within the same clinical setting, reducing the risk of altered hemodynamic conditions or altered patient stress levels, increasing statistical confidence.

## Conclusion

Assessment of LVEF and SV with HAND is both challenging and promising. Our results suggest interchangeability between HAND and SE for both LVEF and SV quantification, and also between HAND and 3DE for LVEF quantification. Therefore, HAND shows to be a promising future tool for LVEF quantification and monitoring of SV in point of care settings. Additional studies investigating the application of HAND at different hemodynamic conditions are needed to qualify HAND as a potential monitoring device in critical care.

## Data Availability

The datasets used and analyzed during the current study are available from the corresponding author on reasonable request.

## Abbreviations

2DE: Two-dimensional echocardiography
3DE: Three-dimensional echocardiography
A2CH: Apical two chamber
A4CH: Apical four chamber
AS: Auto strain
CV: Coefficient of variation
DHM: Dynamic heart model
DICOM: Digital imaging and communication in medicine
HAND: Handheld ultrasound device
ICC-agreement: Intra class correlation of agreement
ICC-techniques: Inter class correlation of agreement between techniques
LOA: Limits of agreement
LV: Left ventricle
LVEF: Left ventricle ejection fraction
RC: Repeatability coefficient
SD: Standard deviation
SE: Standard echocardiography
SV: Stroke volume
